# Post-TAVR patients with atrial fibrillation: are NOACs better than VKAs?—A meta-analysis

**DOI:** 10.3389/fcvm.2023.1175215

**Published:** 2023-08-31

**Authors:** Lu Wang, Wanyue Sang, Yi Jian, Xiaoxue Zhang, Yafan Han, Feifei Wang, Liang Wang, Suxia Yang, Subinuer Wubulikasimu, Li Yang, Huaxin Sun, Yaodong Li

**Affiliations:** ^1^ Cardiac Pacing and Electrophysiology Department, The First Affiliated Hospital of Xinjiang Medical University, Urumqi, China; ^2^ Xinjiang Key Laboratory of Cardiac Electrophysiology and Cardiac Remodeling, The First Affiliated Hospital of Xinjiang Medical University, Urumqi, China

**Keywords:** transcatheter aortic valve replacement, atrial fibrillation, anticoagulant therapy, new oral anticoagulants, vitamin K antagonists

## Abstract

**Objective:**

This study aimed to compare the efficacy of novel oral anticoagulants (NOACs) with traditional anticoagulants vitamin K antagonists (VKAs) in patients with atrial fibrillation (AF) post transcatheter aortic valve replacement (TAVR).

**Methods:**

Studies comparing the usage of NOACs and VKAs in AF patients with oral anticoagulant indication post-TAVR were retrieved from PubMed, EMBASE, Medline, and Cochrane databases from their building-up to Jan. 2023. The literature was screened in line of inclusion and exclusion criteria. Risk ratio (RR) or odds ratio (OR),95% confidence interval (CI) and number needed to treat (NNT) were calculated for four main indexes that composite endpoints composed mainly of any clinically relevant risk events, stroke, major bleeding, and all-cause mortality. Subsequently, a meta-analysis was performed using the RevMan5.3 and Stata 16.0 software.

**Results:**

In the aggregate of thirteen studies, contained 30388 post-TAVR patients with AF, were included in this meta-analysis. Our results indicated that there was no significant difference in stroke between the NOACs group and the VKAs group, and the NOACs group had a numerically but non-significantly higher number of composite endpoint events compared with the other group. Nevertheless, the incidence of major bleeding [11.29% vs. 13.89%, RR 0.82, 95%CI (0.77,0.88), *P* < 0.00001, *I*² = 69%, NNT = 38] and all-cause mortality [14.18% vs. 17.61%, RR 0.83, 95%CI (0.79,0.88), *p* < 0.00001, *I*² = 82%, NNT = 29] were significantly lower in the NOACs group than another group.

**Conclusion:**

Taken together, our data indicated that the usage of NOACs reduced the incidence of major bleeding and all-cause mortality compared to VKAs in post-TAVR patients with AF.

## 1. Introduction

Transcatheter aortic valve replacement (TAVR), also referred to as transcatheter aortic valve implantation; TAVI), has significantly reduced the rate of death in patients with severe aortic stenosis compared with surgical aortic valve replacement (SAVR) (1). Therefore, it is an ideal treatment option for patients with severe aortic stenosis, with higher surgical risk or contraindications (2). Atrial fibrillation (AF) is a common tachyarrhythmia after TAVR, which can be divided into pre-existing AF and new-onset AF. Pre-existing AF refers to the AF before TAVR, or the presence of AF during admission or operation. The incidence of pre-existing AF in TAVR patients has been reported as high as 49% (3). New-onset AF refers to new AF after TAVR, Sannino et al.(4) found that there are 60% of patients with new-onset AF after TAVR through Meta analysis. In addition, AF events significantly increase the risk of stroke, bleeding, and death after TAVR (5, 6). Therefore, there is need for anticoagulant therapy for such patients.

Currently, the common anticoagulants in clinical practice mainly include vitamin K antagonists (VKAs) and new oral anticoagulants (NOACs). The NOACs include factor Xa inhibitors such as rivaroxaban, apixaban or edoxaban, and factor Ⅱa inhibitors such as dabigatran. Data from studies on anticoagulation treatment of post-TAVR patients with AF demonstrated the superiority of the efficacy of NOACs compared to that of VKAs (7). However, due to insufficient evidence, there is still no consensus among clinicians on the use of anticoagulation treatment in these patients. Here, we performed a meta-analysis to determine and compare the efficacy of NOACs and VKAs in post-TAVR patients with AF.

## 2. Materials and methods

### 2.1. Search strategies

This study was guided by the Preferred Reporting Items for Systematic Reviews and Meta-Analyses guidelines (8). A comprehensive search of PubMed, Cochrane, EMBASE, Medline and other databases (until January 2023) was conducted to identify all eligible trials and primary references. The following search terms were used: ((oral anticoagulation) OR (oral anticoagulant*) OR (OAC*) OR (VKA*) OR (warfarin) OR (DOAC*) OR (NOAC*) OR (Dabigatran) OR (Apixaban) OR (Rivaroxaban) OR (Edoxaban)) AND ((transcatheter aortic valve) OR (TAVR) OR (TAVI)) AND ((atrial fibrillation) OR (AF)) AND (trial). In addition, to fill in any omission, we manually searched the references in all reviews in related fields for other relevant studies, which yielded a total of 357 studies.

### 2.2. Inclusion and exclusion criteria

We used EndNoteX9, the literature management software, to manage and avoid duplicate studies. Two researchers independently reviewed the title and abstract of the articles following the inclusion and exclusion criteria. In case of a disagreement, a third researcher re-read the articles and reached a conclusion. When a highly relevant article was encountered during the review process, the full text was read in detail to determine whether it met the inclusion criteria.

#### 2.2.1. Inclusion criteria

This study included randomized controlled trials (RCT) or observational studies such as cohort or case-control studies, which compared the anticoagulation effects of NOACs and VKAs in post-TAVR patients with AF. The studies or trials with any of the following end points: composite endpoints of any related clinical risk events (death from all causes, myocardial infarction, stroke, systemic thromboembolism, valvular thrombosis, or hemorrhage), all-cause mortality, major bleeding, or stroke were included. In addition, the included studies' patients >18 years old.

#### 2.2.2. Exclusion criteria

We excluded studies where oral anticoagulants were not used, conference abstracts, case reports, review articles and animal experiments. Besides, studies whose data could not be extracted and those with patients at high blood risk we also excluded from the analysis.

### 2.3. Data extraction

Two researchers independently extracted basic information in the studies, such as title, author's name, country of publication, and race as well as baseline characteristics such as sample size, gender composition, mean age, and duration of follow-up. The researchers also extracted details of the usage of NOACs and antiplatelet drugs post-TAVR as well as study end points, which included composite endpoints consisting of any relevant clinical adverse events, all-cause mortality, major bleeding or stroke. Data extraction was conducted by mutual negotiation, and all disagreements were resolved by consensus with the third researcher.

### 2.4. Quality evaluation and statistics

The quality of each study was assessed using the Cochrane collaboration tool (9). Meta-analysis was performed using RevMan5.3 and Stata 16.0, while the random effects model was employed to calculate RR and 95% confidence interval (CI). *P* < 0.05 was considered statistically significant. Cochran's q test and heterogeneity test (*I*²) were also performed. If *I*² < 50% indicated low heterogeneity, fixed-effect model was adopted while if *I*² > 50% indicated high heterogeneity, the random effects model was employed to analyze the data (10). Absolute risk reduction (ARR) was calculated by subtracting the events rate of the NOACs group to VKAs treated patients, and then we calculated the NNT using the formula NNT = 1/ ARR. Publication bias was tested using funnel plots when more than 10 studies were included in the analysis.

## 3. Results

### 3.1. Search results and study characteristics

**Figure 1:**
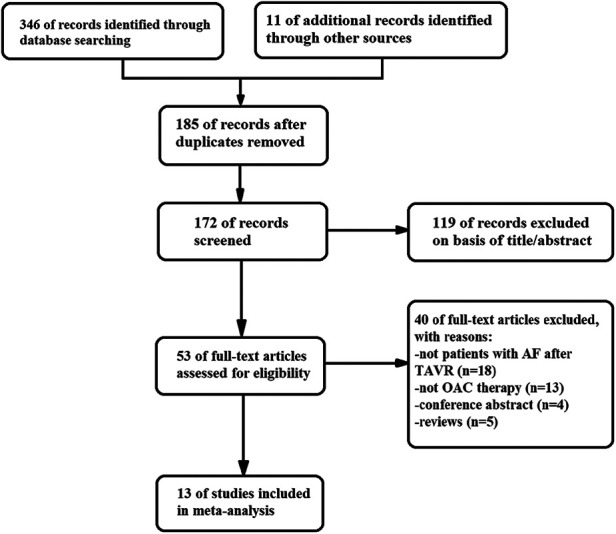
Flow diagram showing the literature search and study selection.

**Figure 2:**
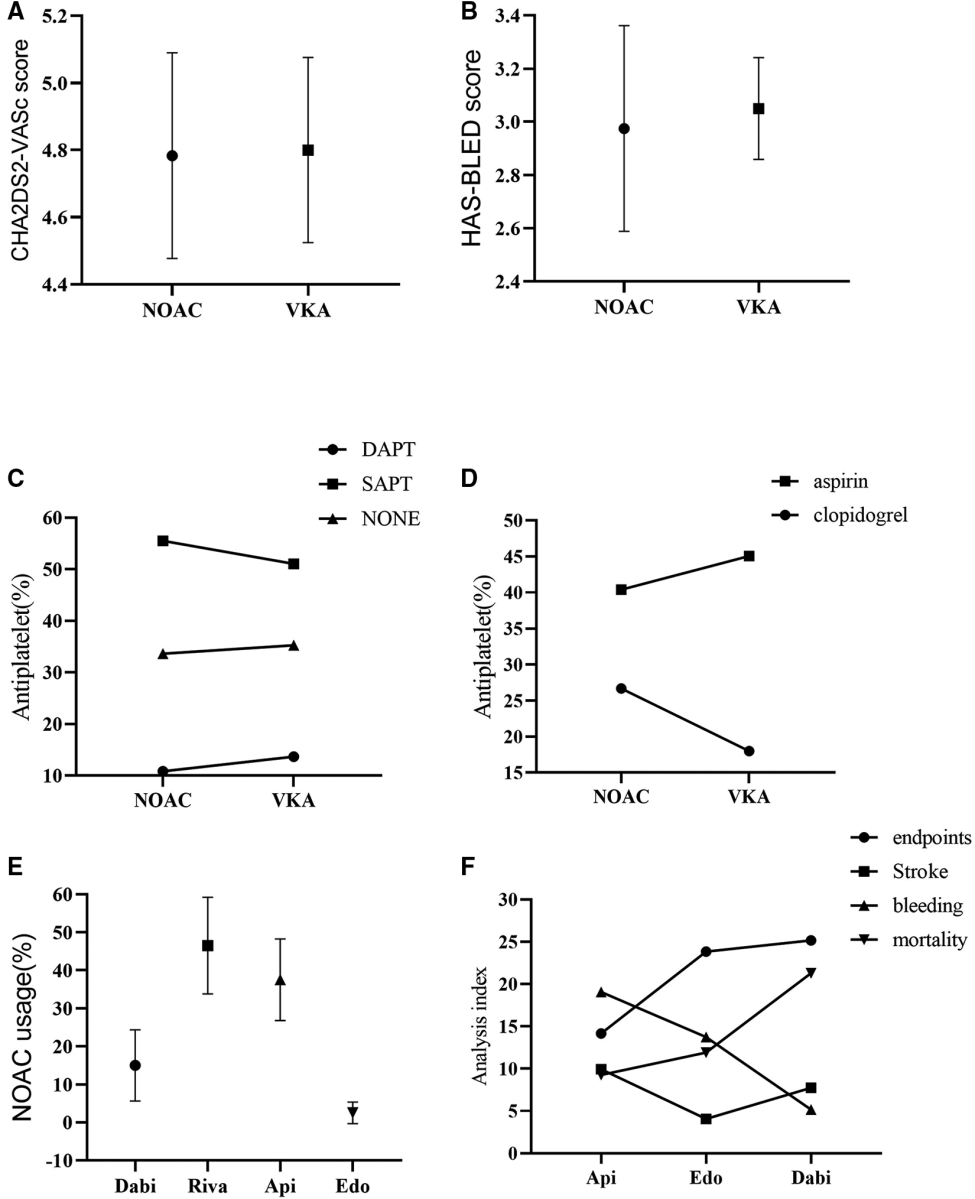
Subgroup analysis. (**A**): CHA2DS2-VASc score; (**B**): HAS-BLED score; (**C**): Combination of antiplatelet drugs; (**D**): Drug usage of SAPT; (**E**): Drug usage of NOAC; (**F**): Analysis of indexes of different NOAC drug therapy; NOAC, novel oral anticoagulant; VKA, vitamin K antagonist; SAPT, single drug antiplatelet therapy; DAPT, double drug antiplatelet therapy; Dabi, dabigatran; Riva, rivaroxaban; Api, apixaban; Edo, edoxaban.

Out of the 357 literatures retrieved in this study, thirteen met the inclusion criteria (7, 11–22). The literature retrieval process is displayed in Figure 1, the baseline characteristics of the included studies are displayed in Table 1, while the details of the usage of NOACs and antiplatelet drugs post-TAVR are shown in Figure 2 and Table 2. The included literatures included 3 RCTs and 10 observational studies containing 30,388 post-TAVR patients with AF, which were published before January 2023. Eleven out of the 13 retrieved literatures reported the primary composite endpoints.

**Table 1:** Characteristics of included studies.

Author	Year	Race	Design	Group	Sample size	Age (y)	Men (%)	BMI	CHA2DS2-VASc score	HAS-BLED score	Composite endpoints (*n*)	Stroke(*n*)	Major bleeding (*n*)	All-cause mortality (*n*)	Follow-up(m)
Butt	2021	White	RC	NOACs	219	83.0 ± 1.2	53.9	NA	5.0 ± 1.4	3.4 ± 0.9	NA	8	11	15	12.0 ± 1.0
				VKAs	516	82.0 ± 1.3	53.7		4.9 ± 1.3	3.3 ± 1.0		14	28	54	27.4 ± 1.0
Collet	2018	White	RCT	NOACs	223	82.3 ± 6.1	49.8	28.3 ± 6.4	4.7 ± 1.4	NA	49	28	21	23	12.0
				VKAs	228	82.5 ± 6.3	54.8	27.9 ± 5.7	4.6 ± 1.4		50	27	27	23	
Didier	2021	White	RC	NOACs	1,378	83.4 ± 6.1	52.6	27.1 ± 5.5	NA	NA	NA	29	55	161	13.0 ± 2.4
				VKAs	1,093	83.5 ± 6.4	51.9	26.9 ± 5.1				37	91	263	21.0 ± 3.4
Geis	2018	White	RC	NOACs	154	83.1 ± 5.3	49.4	26.6 ± 5.3	4.6 ± 1.2	2.7 ± 0.8	17	5	3	12	6.0
				VKAs	172	83.0 ± 4.9	45.3	27.0 ± 5.3	4.8 ± 1.3	2.9 ± 0.8	14	2	3	11	
Jochheim	2019	White	PC	NOACs	326	81.6 ± 6.7	47.9	26.3 ± 5.2	NA	NA	63	10	69	47	12.0
				VKAs	636	81.1 ± 6.1	47.3	26.6 ± 4.9			87	13	146	70	
Kalogeras	2020	White	RC	NOACs	115	81.9 ± 6.3	59.1	27.3 ± 5.8	NA	NA	13	NA	7	13	15.1 ± 3.8
				VKAs	102	82.5 ± 5.8	57.8	25.9 ± 5.8			16		4	16	
Kawashima	2020	Asian	PC	NOACs	227	84.4 ± 4.7	30.4	22.6 ± 3.8	5.1 ± 1.0	2.6 ± 0.8	23	NA	NA	23	19.0 ± 2.5
				VKAs	176	84.3 ± 4.9	36.9	21.7 ± 3.7	5.2 ± 1.1	2.9 ± 0.9	41			41	
Kosmidou	2019	White	RC	NOACs	155	82.8 ± 6.7	65.6	28.4 ± 6.1	5.6 ± 1.3	NA	39	12	8	33	33.6 ± 3.6
				VKAs	778						234	41	43	207	
Mangner	2019	White	RC	NOACs	182	80.0	48.9	27.7	5.7	3.3 ± 0.7	24	2	20	1	1.0
				VKAs	117		47.9	27.8			23	2	14	4	
Montalescot	2022	Multiple	RCT	NOACs	370	81.5 ± 6.1	54.1	27.4 ± 5.4	4.3 ± 1.4	NA	35	31	92	32	12.0
				VKAs	392	82.3 ± 6.3		27.1 ± 4.7	4.4 ± 1.4		27	23	91	25	
Seeger	2017	White	PC	NOACs	141	82.1 ± 5.3	49.6	27.2 ± 4.2	5.0 ± 1.2	3.2 ± 1.1	22	1	NA	19	12.0
				VKAs	131	80.5 ± 6.3	51.9	27.4 ± 5.1	4.9 ± 1.1	3.1 ± 1.1	9	1		6	
Tanawuttiwat	2022	Multiple	RC	NOACs	8,127	83.0	56.9	27.6	3.0	NA	204	204	967	1,284	12.0
				VKAs	13,004	84.0	56.6	27.5			308	308	1,951	2,367	
Van Mieghem	2021	Multiple	RCT	NOACs	713	82.1 ± 5.4	51.3	27.5 ± 5.7	4.5 ± 1.4	NA	170	29	98	85	18.5
				VKAs	713	82.1 ± 5.5	53.6	27.9 ± 5.4	4.5 ± 1.3		157	35	68	93	17.7

RC, retrospective cohort; RCT, randomized controlled trial; PC, prospective cohort; NOACs, novel oral anticoagulants; VKAs, vitamin K antagonists; NA, not applicable.

**Table 2:** Usage of NOACs and antiplatelet drugs.

Author	Butt		Collet		Didier		Geis		Jochheim	
	NOACs	VKAs	NOACs	VKAs	NOACs	VKAs	NOACs	VKAs	NOACs	VKAs
NONE	Dabi:30.6% Riva:31.1% Api:38.3%	Warf	Api	Warf	Dabi:12.1% Riva:35.4% Api:52.5%	Warf	Dabi:9.1% Riva:51.3% Api:35.1% Edo:4.5%	Warf	Dabi:7.1% Riva:53.7% Api:39.2%	Warf
Aspirin	64.8%	60.5%	25.6%	22.8%	45.7%	45.5%	NA	NA	52.5%	53.3%
Clopidogrel					8.1%	8.5%			6.4%	9.9%
DAPT	18.3%	16.1%	2.2%	1.3%	10.2%	9.2%			23.0%	20.3%
Author	Kalogeras		Kawashima		Kosmidou		Mangner		Montalescot	
	NOACs	VKAs	NOACs	VKAs	NOACs	VKAs	NOACs	VKAs	NOACs	VKAs
NONE	Dabi Riva Api Edo	Warf	Dabi Riva Api Edo	Warf	Dabi	Warf	Dabi:16.0% Riva:61.0% Api:22.5% Edo:0.5%	Warf	Api	Warf
Aspirin	9.3%	26.4%	60.4%	67.0%	57.4%	58.5%	NA	NA	24.9%	5.9%
Clopidogrel	72.1%	35.8%								
DAPT	17.4%	37.7%	3.5%	8.0%					2.2%	0.5%
Author	Seeger		Tanawuttiwat		Van Mieghem					
	NOACs	VKAs	NOACs	VKAs	NOACs	VKAs				
NONE	Api	Warf	Dabi:8.8% Xa inhibitor:91.2%	Warf	Edo	Warf				
Aspirin	NA	NA	54.1%	55.0%	46.0%	50.4%				
Clopidogrel			20.1%	17.8%						
DAPT			10.1%	16.3%						

NOACs, novel oral anticoagulants; VKAs, vitamin K antagonists; DAPT, double drug antiplatelet therapy; Dabi, dabigatran; Riva, rivaroxaban; Api, apixaban; Edo, edoxaban; Warf, warfarin; NA, not applicable.

### 3.2. Study quality and data synthesis

**Figure 3:**
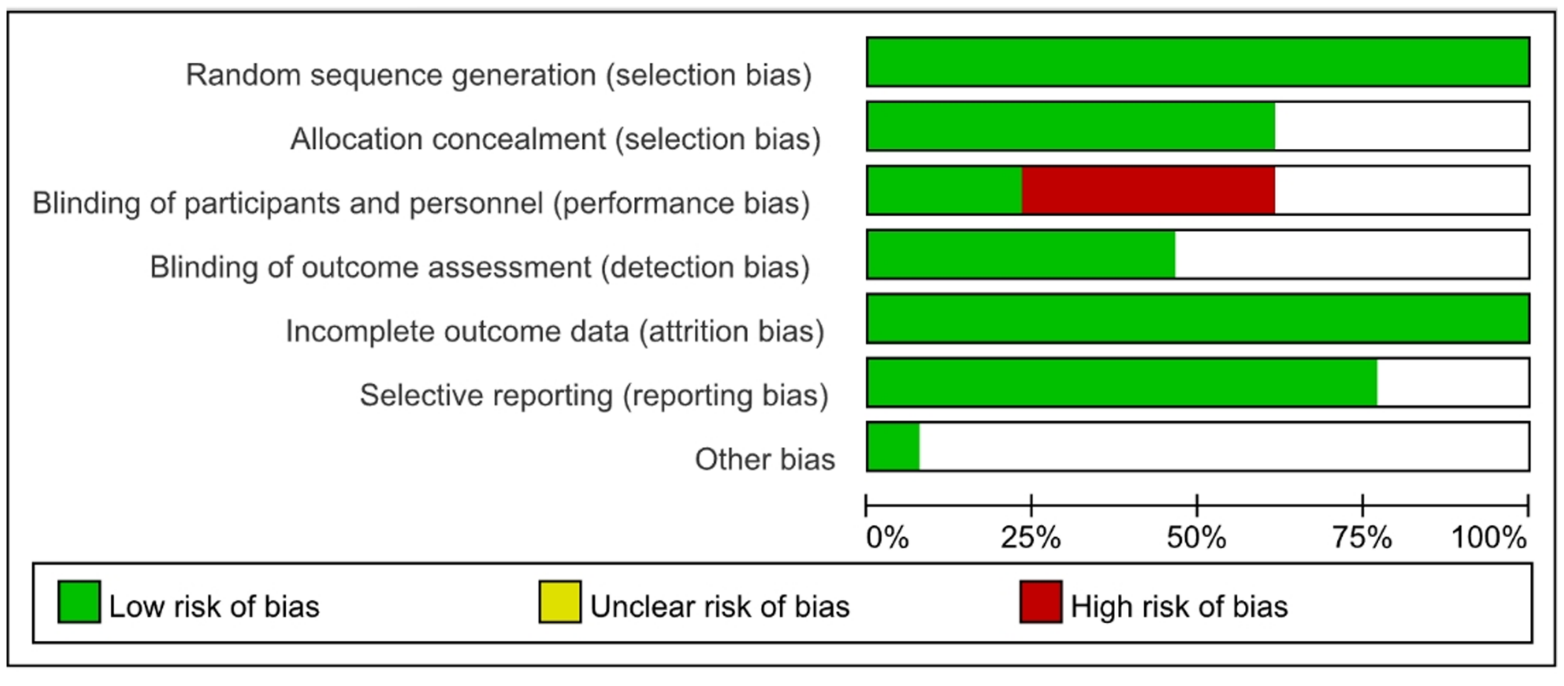
Quality evaluation.

The quality of the included literatures was analyzed as shown in Figure 3.

**Figure 4:**
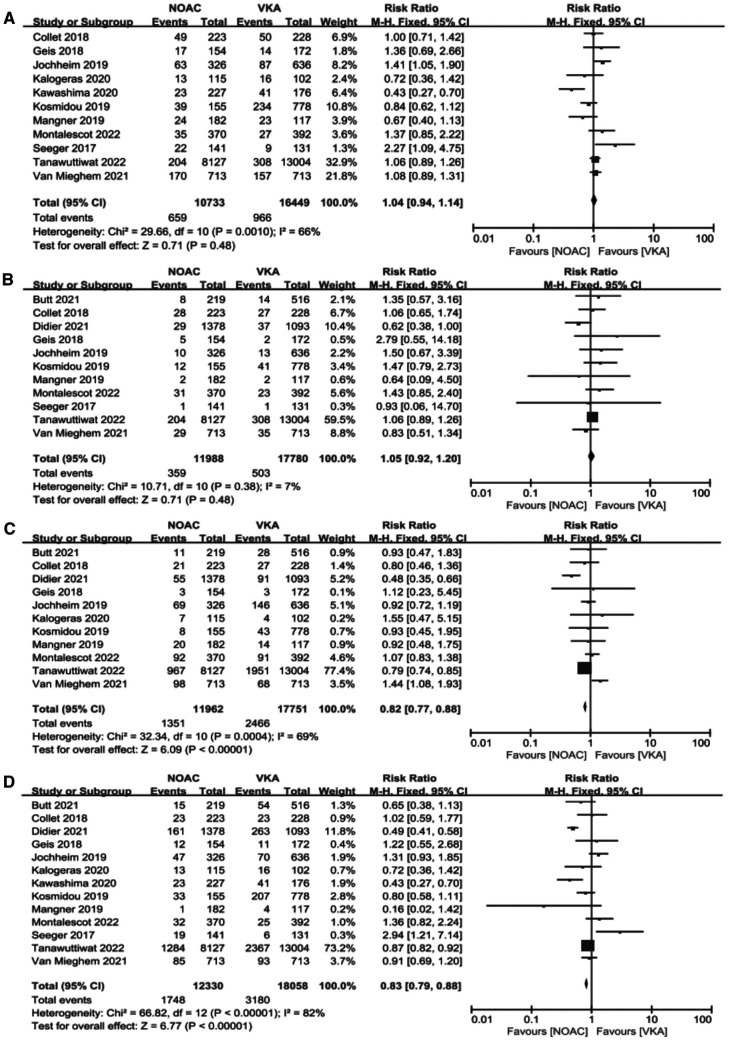
Forest plot. (**A**): composite endpoints; (**B**): stroke; (**C**): major bleeding; (**D**): all-cause mortality; NOACs, novel oral anticoagulant; VKA, vitamin K antagonist; CI, confidence interval.

We performed meta-analysis of the 11 studies which reported composite endpoints using random-effects models. The data showed that the incidence of major bleeding [11.29% vs. 13.89%, RR 0.82, 95%CI (0.77, 0.88), *p* < 0.00001, *I*² = 69%, NNT = 38] and all-cause mortality [14.18% vs. 17.61%, RR 0.83, 95%CI (0.79, 0.88), *p* < 0.00001, *I*² = 82%, NNT = 29] were significantly lower in the NOACs group compared to those in the VKAs group. Nevertheless, the NOACs group had a numerically but non-significantly higher number of composite endpoint events compared with the other group [6.14% vs. 5.85%, RR 1.04, 95%CI (0.94, 1.14), *p* = 0.48, *I*² = 66%], and there was no prominent difference in the incidence of stroke after comparing the two groups [2.99% vs. 2.83%, RR 1.05, 95%CI (0.92, 1.20), *p* = 0.48, *I*² = 7%] (Figure 4).

#### 3.2.1. Subgroup analysis

**Figure 5:**
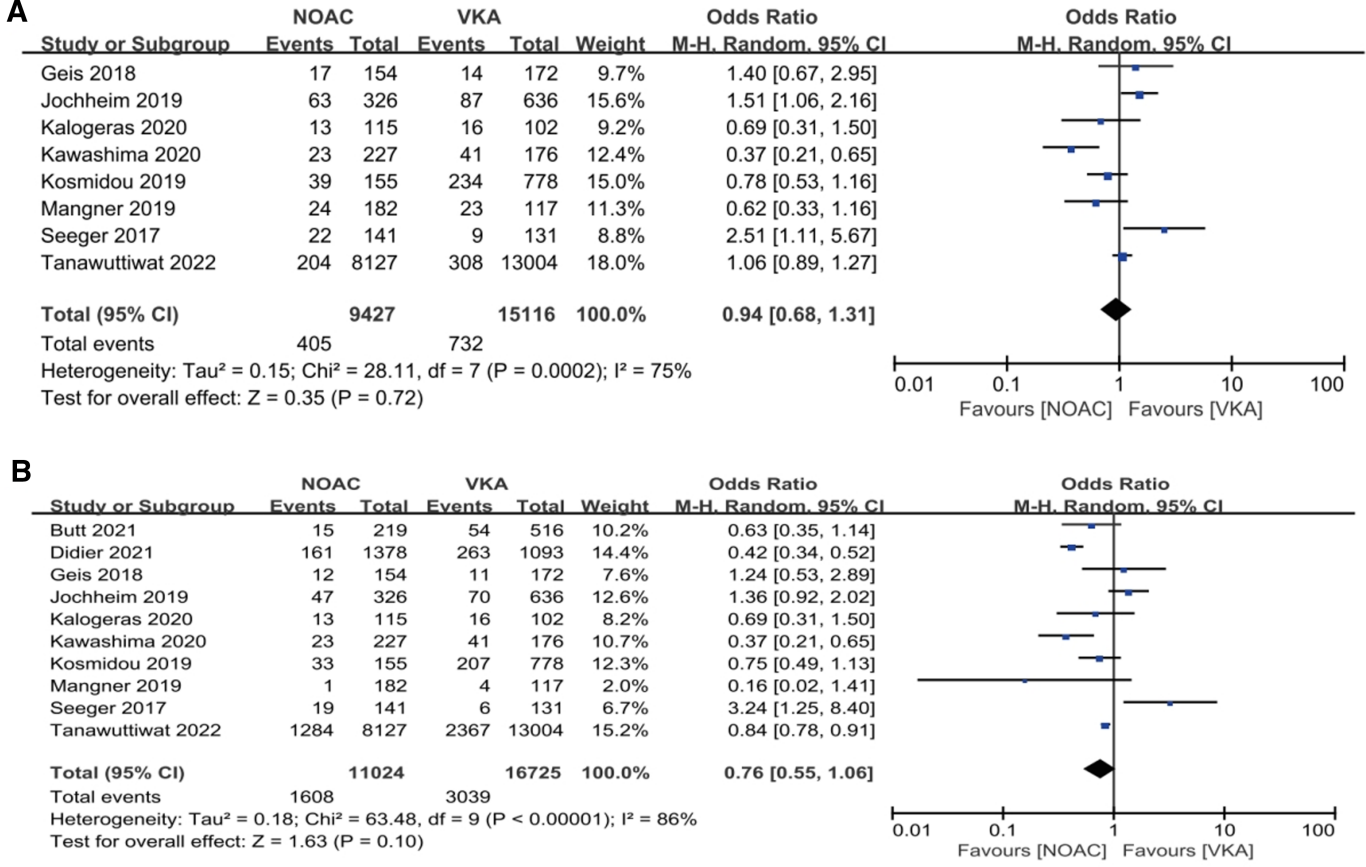
Subgroup analysis. (**A**): composite endpoints; (**B**): all-cause mortality; NOAC, novel oral anticoagulant; VKA, vitamin K antagonist; CI, confidence interval.

Our analysis showed that in the observational study group, there was no significant reduction in composite endpoints incidence [4.30% vs. 4.84%, OR 0.94, 95%CI (0.68,1.31), *p* = 0.72, *I*² = 75%] and all-cause mortality [14.59% vs. 18.17%, OR 0.76, 95%CI (0.55,1.06), *p* = 0.10, *I*² = 86%] in the NOACs group compared with the other group (Figure 5).

In addition, the data showed that CHA2DS2VASc score (4.80 vs. 4.78, *p* = 0.92) and HAS-BLED score (3.05 vs. 2.98, *p* = 0.74) were slightly lower in the NOACs group than the VKAs group (Figures 2A,B, Table 2).

On the other hand, combined single drug antiplatelet therapy (SAPT) yielded more effect than single anticoagulant therapy in both groups, while combined double drug antiplatelet therapy (DAPT) was least (53.28 vs. 34.46 vs. 12.27, *p *< 0.0001). Meanwhile, aspirin conferred more effect compared to clopidogrel in SAPT (42.73 vs. 22.34, *p* = 0.06) (Figures 2C,D, Table 2).

Analysis of the usage of NOACs drugs showed that rivaroxaban was the most used, followed by apixaban and dabigatran, while edoxaban was the least (46.50 vs. 37.52 vs. 14.98 vs. 1.00, *p *< 0.0001). Nevertheless, the incidence of adverse events was slightly lower with apixaban than edoxaban, but higher with dabigatran (12.95 vs. 13.38 vs. 14.85, *p* = 0.94) (Figures 2E,F, Table 2). The incidence of adverse events associated with the use of rivaroxaban could not be analyzed because there were no studies that used rivaroxaban alone.

### 3.3. Publication bias and sensitivity analysis

**Figure 6:**
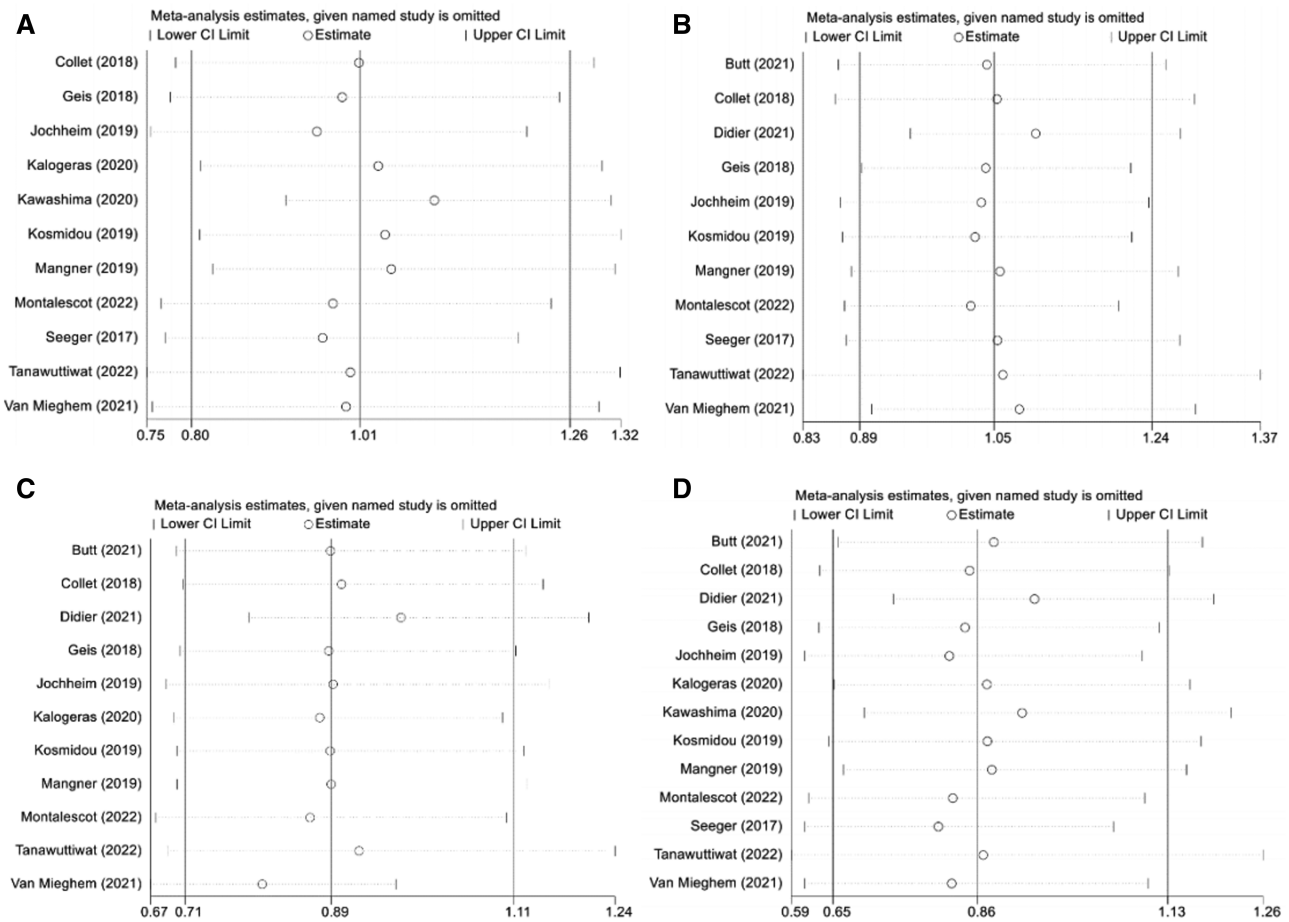
Sensitivity analysis. (**A**): composite endpoints; (**B**): stroke; (**C**): major bleeding; (**D**): all-cause mortality; NOAC, novel oral anticoagulant; VKA, vitamin K antagonist; CI, confidence interval.

As shown in the funnel plot (Supplementary Figure S1), studies are evenly distributed on both sides of the line, basically symmetrical, and large sample studies are distributed at the top of the funnel. Simultaneously, Egger's test also indicated lack of significant publication bias (*p* > 0.05). Sensitivity analysis was performed to determine the primary source of heterogeneity, and after omitting all studies one by one, the OR and 95%CI intervals remained aligned and in a similar range (Figure 6). Once again, we conducted a sensitivity analysis including only data from the 3 RCTs and the similar result is obtained (Supplementary Figure S2). Therefore, the analysis results can be considered stable and reliable.

## 4. Discussion

TAVR is now extensively used in high-risk patients with aortic stenosis (23). Nevertheless, the incidence of postoperative AF remains high, superimposing the risk of thromboembolism. Therefore, the usage of anticoagulant therapy in these type of patients remains a significant issue. The 2020 ACC and AHA guidelines (24) advise that patients at high-risk of major bleeding post-TAVR (CHA2DS2-VASc score: ≥3 for women and ≥2 for men) should be firstly treated with DAPT for six months, meanwhile patients at low risk of major bleeding should be firstly treated with oral anticoagulants for three months, after that, lifelong use of SAPT. Yet, detailed recommendations in the guidelines with regard to oral anticoagulation still imprecision, and the usage of NOACs in post-TAVR patients with AF continues to be controversial (25). Based on this, the study aims to explore the efficacy of NOACs and VKAs in post-TAVR patients with AF, so as to provide evidence for clinical medication.

In a previous meta-analysis, Li et al. (26) analyzed 10 studies with a total of 10,563 TAVR patients with AF, and the results demonstrated that there was no significant difference in the incidence of stroke after comparing the two groups [HR1.20, 95%CI (0.71, 2.04), *p* = 0.50], which was in accordance with our results. This may be because TAVR patients are older, have more complications, and have a higher risk of non-AF related stroke (27). Oliveri et al. (28) performed a meta-analysis of 29,485 TAVR patients with AF in ten literatures and indicated that all-cause mortality was lower in the NOACs group compared with the VKAs group [RR0.90; 95% CI (0.81,0.99), *p* = 0.04]. In addition, we also found that all-cause mortality was lower in the NOACs group than in the VKAs group, which is consistent with previous findings. However, compared to previous meta-analyses, our study for the first time found a lower incidence of major bleeding in the NOACs group, suggesting a lower risk of hemorrhoea in the NOAC group, which may be related to the fact that: Compared with the VKAs group, the CHA2DS2-VASc and HAS-BLED scores in the NOACs group were slightly lower, so anticoagulants were used less. In the meantime, low NNT of major bleeding and all-cause mortality means that less clinical effort is required to produce a favorable result in post-TAVR patients with AF who use NOACs. Nevertheless, in subgroup analysis of randomized controlled trials and observational studies, four main indexes indicated no significant statistical differences. This maybe because there are few randomized controlled studies and most of the included studies were retrospective, which have inherent limitations compared to prospective studies. Additionally, this is also the main source of heterogeneity in this study. Furthermore, the results of sensitivity analysis possibly due to lacking of power in the smaller sample or bias in the non-RCTs with more favorable results and publication bias (though not confirmed in the funnel plots). Combined with the above indicators, the results of this study found that compared with VKAs, the use of NOACs for anticoagulation therapy in post-TAVR patients with AF is generally beneficial.

In addition, we conducted an in-depth analysis of the selection of NOACs combined with antiplatelet drugs in post-TAVR patients with AF. The findings showed that rivaroxaban was the most used NOAC drug, followed by apixaban and dabigatran, while edoxaban was the least used. Hayat et al. (29) conducted a real-world study of 668 patients with AF which showed that rivaroxaban had significantly better compliance and lower bleeding risk than dabigatran and Apoxaban. Therefore, Rivaroxaban has become the most popular new oral anticoagulant drug in clinic due to its once-daily dosing schedule and better anticoagulant effect. In terms of the use of combined antiplatelet drugs, the analysis showed that combined SAPT yielded more effect than anticoagulant therapy alone, while combined DAPT conferred the least effect. It may be due to the patients have more complications. To sum up, most clinicians are more inclined to choose the regimen of rivaroxaban combined with aspirin. It is speculated that this regimen has relatively few side effects on patients, so patients' compliance is better. In addition, it may also be due to clinical experience which indicate that this regimen is more conducive to the prognosis of patients.

Analysis of the literatures included in this study indicated that AF patients with perioperative anticoagulation mostly stopped VKAs nearly seven days pre-TAVR, whereas NOACs were stopped nearly 2 days pre-TAVR, whereupon, resumed oral anticoagulation 12–48 h post-TAVR. In addition, our research shows that, in the choice of drug dosage, the habituation of most DOACs was identical. For instance, apixaban 5 mg, bid; 2.5 mg together with antiplatelet therapy, bid. But the dosage of rivaroxaban is different under distinct conditions. When patients' creatinine clearance (CrCl) ≥50 ml/min, rivaroxaban was given 15 mg/d in Japan, and 20 mg/d in Europe or the United States. When patients' CrCl 30–49 ml/min, rivaroxaban was given 10 mg/d in Japan, while 15 mg/d in Europe and the United States (30, 31). Given the widespread use of rivaroxaban in post-TAVR patients with AF, there is a need for larger studies to harmonize the dosage. In addition, this study showed that different countries have similar dosing choices for antiplatelet drugs: Patients receiving combined SAPT take 75–100 mg of aspirin daily, whereas clopidogrel 75 mg/d is added only when there is a necessary coronary indication for DAPT.

Combined with the findings of the present study and the overall state of the available evidence, the use of NOACs is an effective and safe anticoagulant strategy. In particular, factor Xa inhibitors (rivaroxaban, edoxaban, and apixaban) have certain antiplatelet effects (32), and may be more suitable for patients with anticoagulant and antiplatelet needs. Although dabigatrun, a direct Ⅱa inhibitor, had little effect on platelet aggregation, its P2Y12 reaction unit was significantly higher than that of factor Xa inhibitors (32), which may be more suitable for patients requiring anticoagulation alone. As a consequence, there are more need to expand the scale of the research to standardize medication regimens in order to achieve an optimal anticoagulant effect.

Additionally, the left atrial appendage is the main source of thrombosis in patients with AF (about 90%). Therefore, left atrial appendage closure (LAAC) can achieve the effect of anticoagulant therapy at the source (33). It has been shown that, currently, it is more common to choose LAAC post-TAVR, but there are also a few reports on the feasibility of “one-stop” surgery than TAVR combined with LAAC (34), and these reports all show the safety, feasibility and effectiveness of LAAC in TAVR patients with AF. Although “one-stop” surgery has been shown to be safe and effective in some small exploratory studies, and its ability to reduce all-cause mortality compared with warfarin, larger randomized clinical trials with longer follow-up are needed to confirm its efficacy and safety. The ongoing Watch-TAVR is a multicenter randomized controlled trial (35), designed to compare the efficacy of Watchman left atrial appendage closure therapy with TAVR combined with drug therapy in TAVR patients, is expected to provide stronger evidence for the application of anticoagulant therapy in post-TAVR patients with AF.

## 5. Limitations

Since the heterogeneity between the two groups was high, this study used a random effects model. This was because there are few randomized controlled studies and most of the included studies were retrospective, which have inherent limitations compared to prospective studies. Besides, the specific drugs used in the studies were not the same and there was significant heterogeneity between studies, possibly due to differences in country, ethnicity and type of study. In addition, some studies included a small sample of patients, which was not very representative.

## 6. Conclusions

Taken together, our analysis demonstrated that NOACs have a lower mortality and a lower incidence of major bleeding in patients of AF with oral anticoagulant indications post-TAVR compared with VKAs, indicating that NOACs is a potential alternative to VKAs. However, there is a need for more high-quality randomized controlled clinical trials to confirm this conclusion.

## Data Availability

The original contributions presented in the study are included in the article/**Supplementary Material**, further inquiries can be directed to the corresponding author.
