# Supplementary material for: Post-TAVR patients with atrial fibrillation: are NOACs better than VKAs?—A meta-analysis
Source: Front Cardiovasc Med. 2023 Aug 31;10:1175215. doi: 10.3389/fcvm.2023.1175215 (PMC10501834; doi:10.3389/fcvm.2023.1175215)
Supplement: Supplementary file 1 [file Image1.pdf]

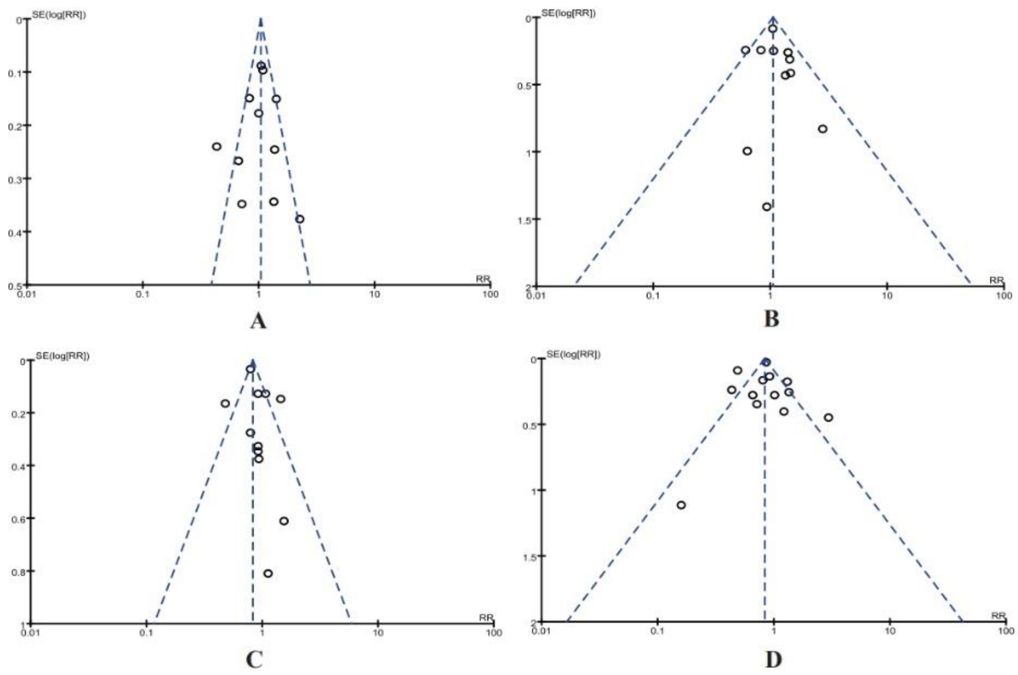

Figure S1. Funnel diagram. **(A)**: composite endpoints; **(B)**: stroke; **(C)**: major bleeding; **(D)**: all-cause mortality; SE: standard error; RR: risk ratio.

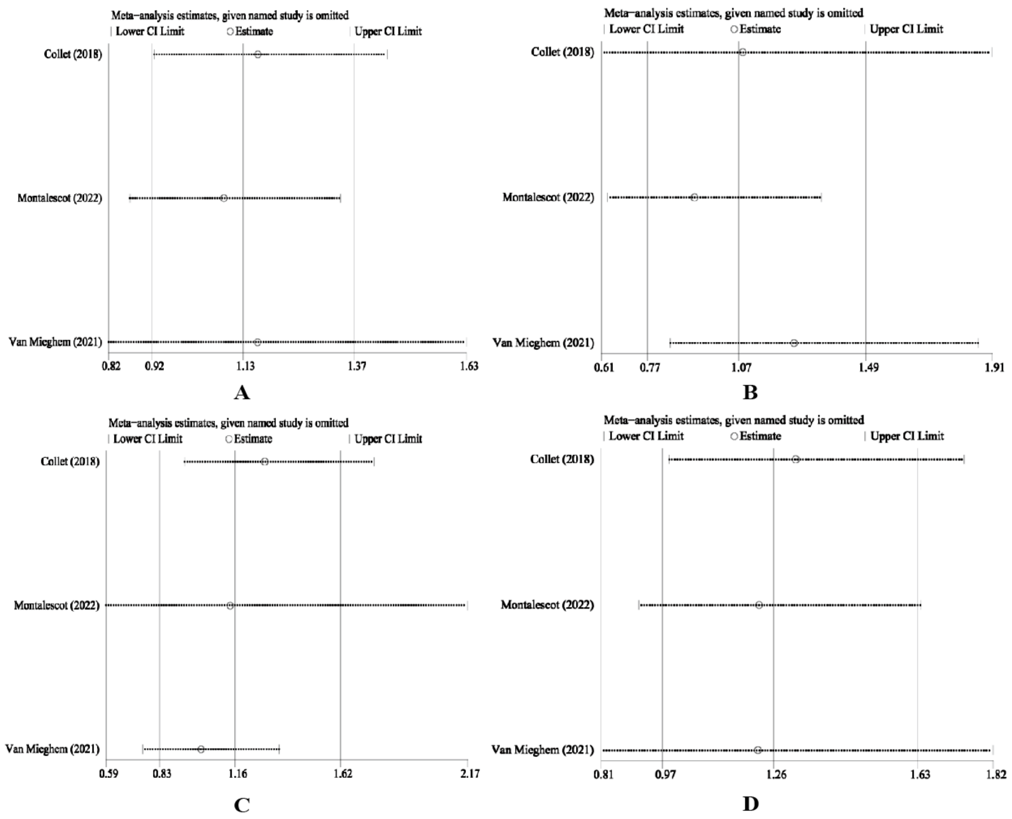

Figure S2. Sensitivity analysis of 3 RCTs. **(A)**: composite endpoints; **(B)**: stroke; **(C)**: major bleeding; **(D)**: all-cause mortality; NOAC: novel oral anticoagulant; VKA: vitamin K antagonist; CI: confidence interval.
